# β-amyloid protein induces mitophagy-dependent ferroptosis through the CD36/PINK/PARKIN pathway leading to blood–brain barrier destruction in Alzheimer’s disease

**DOI:** 10.1186/s13578-022-00807-5

**Published:** 2022-05-26

**Authors:** Jianhua Li, Mengyu Li, Yangyang Ge, Jiayi Chen, Jiamin Ma, Chenchen Wang, Miaomiao Sun, Li Wang, Shanglong Yao, Chengye Yao

**Affiliations:** 1 Department of Anesthesiology, Union Hospital, Tongji Medical College, Huazhong University of Science and Technology, Wuhan, 430022 China; 2 Institute of Anesthesia and Critical Care Medicine, Union Hospital, Tongji Medical College, Huazhong University of Science and Technology, Wuhan, 430022 China; 3 Department of Critical Care Medicine, The First Affiliated Hospital, College of Medicine, Shihezi University, Shihezi, 832000 China; 4 Department of Neurology, Union Hospital, Tongji Medical College, Huazhong University of Science and Technology, Wuhan, 430022 China

**Keywords:** Alzheimer’s disease, Blood–brain barrier, Pericytes, Fatty acid transporter, Beta-amyloid, Mitophagy, Ferroptosis

## Abstract

**Introduction:**

Blood–brain barrier (BBB) dysfunction may occur at the onset of Alzheimer’s disease (AD). Pericytes are a vital part of the neurovascular unit and the BBB, acting as gatekeepers of the BBB. Amyloid β (Aβ) deposition and neurofibrillary tangles in the brain are the central pathological features of AD. CD36 promotes vascular amyloid deposition and leads to vascular brain damage, neurovascular dysfunction, and cognitive deficits. However, the molecular mechanism by which pericytes of the BBB are disrupted remains unclear.

**Objectives:**

To investigate the effect of low-dose Aβ1-40 administration on pericyte outcome and the molecular mechanism of BBB injury.

**Methods:**

We selected 6-month-old and 9-month-old APP/PS1 mice and wild-type (WT) mice of the same strain, age, and sex as controls. We assessed the BBB using PET/CT. Brain pericytes were extracted and cocultured with endothelial cells (bEnd.3) to generate an in vitro BBB model to observe the effect of Aβ1-40 on the BBB. Furthermore, we explored the intracellular degradation and related molecular mechanisms of Aβ1-40 in cells.

**Results:**

BBB permeability and the number of pericytes decreased in APP/PS1 mice. Aβ1-40 increased BBB permeability in an in vivo model and downregulated the expression of CD36, which reversed the Aβ-induced changes in BBB permeability. Aβ1-40 was uptaked in pericytes with high CD36 expression. We observed that this molecule inhibited pericyte proliferation, caused mitochondrial damage, and increased mitophagy. Finally, we confirmed that Aβ1-40 induced pericyte mitophagy-dependent ferroptosis through the CD36/PINK1/Parkin pathway.

**Conclusion:**

PDGFRβ (a marker of pericytes), CD36, and Aβ colocalized in vitro and in vivo, and Aβ1-40 caused BBB disruption by upregulating CD36 expression in pericytes. The mechanism by which Aβ1-40 destroys the BBB involves the induction of pericyte mitophagy-dependent ferroptosis through the CD36/PINK1/Parkin pathway.

**Supplementary Information:**

The online version contains supplementary material available at 10.1186/s13578-022-00807-5.

## Introduction

More than 50% of clinically diagnosed Alzheimer’s disease (AD) patients have vascular alterations; however, the role of age-related vascular factors in the mechanism of AD remains unclear [1]. Before the onset of dementia, neurodegeneration, and/or atrophy, initial blood–brain barrier (BBB) impairment and/or dysfunction can be observed in AD [2–5]. Articularly at the capillary level, pericytes play a vital role in the formation and maintenance of the BBB [6–8]. Pericytes degenerate in individuals with AD and play a crucial role in the BBB clearance of Aβ [9–11]. Pericyte death occurs when Aβ aggregates in the cell, exceeding the clearance of this protein, or when hypoxia occurs. Transgenic mice deficient in PDGFRβ in pericytes not only have learning and memory impairments but also have enhanced Aβ deposition in the brain and neuronal cell death in the hippocampus and cerebral cortex [12, 13]. Pericytes may transport Aβ across the BBB into the blood [14, 15]. Previously, the role of endothelial cells in the BBB has been extensively researched, but there have been few studies on the role of pericytes in the disruption of the BBB by Aβ, and studies on the changes in pericytes due to Aβ are lacking.

Aβ is formed by amyloid precursor protein (APP) [16], a membrane protein that acts as a signal receptor during neuronal activity. Its production and accumulation in the brains of individuals with AD are considered hallmarks of the amyloid hypothesis [17]. The common soluble monomer subtypes of Aβ are β1-42 (< 10%) and β1-40 (< 80%) [6]. Aβ1-42 has two more hydrophobic amino acids, which easily form insoluble aggregates, leading to neuroimmune inflammation [18–20]. Aβ1-40 is easily deposited in the vascular system [21]. A typical cerebrovascular disease associated with Aβ1-40 deposition is cerebral amyloid angiopathy (Aβ-cerebral amyloid angiopathy, CAA) [22]. The destruction of the BBB is an essential reason for the peripheral clearance of Aβ [23]. This phenomenon not only decreases the release of Aβ but also increases the transport of peripheral Aβ to the center through the BBB, which eventually leads to high levels of intracranial Aβ deposition [24], accelerating disease progression. The mechanism of Aβ-induced BBB destruction is still unclear. In fact, Aβ is the initiating factor of BBB destruction. Autopsy results showed apparent BBB destruction around blood vessels with Aβ deposition. Therefore, elucidation of the mechanism of Aβ injury to the BBB will help maintain the integrity of the BBB and decrease Aβ deposition in the central nervous system.

Fatty acid transporter, also known as scavenger receptor cluster 36 (CD36), is a multifunctional glycoprotein involved in apoptotic cell uptake, signal transduction, cell adhesion, angiogenesis, and immune function [25, 26]. This molecule is essential for Aβ1-40-induced vascular oxidative stress and neurovascular dysfunction. CD36 can also change its structure to uptake and absorb cellular contents such as oxidized phospholipids, apoptotic cells, and amyloid [27, 28]. Previous studies have confirmed that the lack of CD36 can offset the cerebrovascular dysfunction of Tg2576 mice and partially normalize the number and morphology of pericytes [29]. However, its expression and mechanism after low-dose Aβ1-40 stimulation of pericytes are still unclear. In particular, the role of CD36 in pericytes in the BBB may become a new molecular target for the treatment of AD-related amyloid angiopathy.

## Materials and methods

### Animals

6-month-old and 9-month-old APP1/PS1 double transgenic AD model mice (9 each) [provided by Jiangsu Changzhou Cavens Experimental Co., Ltd. (license number: SCXK (Su) 2016-0010)] and 9 wild-type (WT) mice (C57BL/6 J) were used [provided by Beijing Sibefu Biotechnology Co., Ltd. (license number: SCXK (Jing) 2019-0010)]. The animals were kept in an SPF animal room at a constant temperature (23 °C ± 2 °C), and the conditions included the alternation of white light and dark (8:00 a.m. to 8:00 p.m. white light; 8:00 p.m. to 8:00 a.m. dark) and free access to food and water. The mice used in this experiment were all males, which met the SPF clean-level standard for laboratory animals issued by the Ministry of Health. Male Sprague–Dawley (SD) rats (30–550 g, approximately 3 weeks old) were provided by the Animal Experiment Center of Tongji Medical College, Huazhong University of Science and Technology. During the experiment, raising and obtaining animal materials complied with the relevant rules and regulations of the Animal Management Committee of Huazhong University of Science and Technology and the International Association for Animal Research (IASP) on the management and protection of experimental animals.

### Antibodies and reagents

The following antibodies were used in this study: β-amyloid (20.1) (Santa Cruz Biotechnology); PDGFRβ (Abcam and CST); NG2 (Abcam); LRP1 (Abcam); CD36 (ABclonal); CD31 (CST); GLUT1 (Abcam); CD36 (BD); β-actin (ABclonal); HSP60 (Santa Cruz Biotechnology); Tim23 (ABclonal); LC3B (Sigma–Aldrich); cleaved-caspase 3 (CST); caspase 3 (ABclonal); BAX (ABclonal); BCL2 (ABclonal); GPx4 (Proteintech); xCT (Proteintech); NOX1 (Proteintech); GAPDH (Proteintech); ferritin (Beyotime); and a Mitophagy Antibody Sampler Kit (CST). Aβ1-40 (ChinaPeptides Co., Ltd.), BODZPY 581/591 (Invitrogen), Mito-FerroGreen (Dojindo), LysoTracker Red (Invitrogen), HiLyte Fluor™ 555-Aβ1-40 (Anaspec), FITC-Aβ1-40 (Bachem), and FITC-dextran (Sigma–Aldrich) were also used.

### Mouse brain PET/CT imaging

Abnormal changes in brain 18F fluorodeoxyglucose (18F-FDG) uptake are often used as a sensitive indicator of the BBB [30]. The procedure used was as follows. (1) One day before the experiment, the mice had not fasted from food and water. (2) Gas anesthesia (2% isoflurane) and tail vein injection of 200 ± 10 µCi 18F-FDG were used. (3) Then, scanning was immediately started, and PET was performed first, followed by CT. (4) PET was performed in dynamic mode with head scan for 10 min; CT was performed in normal mode. (5) The reconstruction conditions were as follows: PET, two iterations, 12 subsets, slightly filtered with time division of 1 min*10; CT, FDK, image size of 256, FOV of 1.0. (6) After scanning, the mice were placed in a lead-shielded room, restored to a regular diet, and fed normally after 10 18F half-lives. The mean standardized uptake value (SUV) was calculated using the following formula: mean pixel value with the decay-corrected region-of-interest activity (μCi/kg)/(injected dose [μCi]/weight [kg]).

### Brain sampling

We used 4% sodium pentobarbital [50 mg/kg, intraperitoneal (i.p.)] to anesthetize the mice and then fix them on the operating table. After thorough disinfection with 75% alcohol, tissue scissors were used to cut the skin along the midline of the mouse sternum from bottom to top. The sternum was cut to fully expose the heart. Care was taken to prevent damage the blood vessels. After the diaphragm was carefully broken, toothless long forceps were used. After carefully clamping and fixing the heart, we quickly inserted the perfusion needle from the apex of the heart into the left ventricle, paying attention to the angle and depth of the needle to avoid penetrating the interventricular septum. Then, we cut a small opening in the right atrial appendage with ophthalmological scissors, opened the perfusion valve, infused precooled saline from the left ventricle, and allowed flow out from the right atrial appendage through the large circulation; the outflow was colorless. The lungs, liver, and mucous membranes were whitish. The mice were perfused and fixed with 4% paraformaldehyde at 4 °C for approximately 10 min. Finally, the intact brain tissue was carefully removed and soaked in 4% paraformaldehyde for histological analysis.

### Immunofluorescence of brain slices

Brain slices prepared from the indicated groups of mice were subjected to double immunofluorescence staining. The primary antibodies used in our studies were mouse anti-β-amyloid (20.1) (1:25, Santa Cruz Biotechnology), rabbit anti-PDGFRβ (1:100, Abcam), and rabbit anti-CD36 (1:100 ABclonal). All images were obtained, and colocalization was visualized, using a Nikon confocal microscope.

### Cell isolation, generation, culture, and activation

In this experiment, the bEnd.3 cell line was purchased from ATCC and was passaged once every 3–4 days according to the company’s requirements. Brain pericytes were extracted according to a previous extraction method [31, 32] and improved. Each time, 5 male SD rats at approximately 3 weeks of age were used. The SD rats were from Huazhong provided by the Animal Experiment Center, Tongji Medical College, University of Science and Technology. After anesthetizing the rat (as described previously), we immediately removed the brain. Then, the whole brain was placed in prechilled 75% alcohol for 3–5 min for disinfection, the cerebral cortex was removed, 500 µl each of 0.1% type II collagenase and DNase I (1000 U/ml) was added and the brain tissue was digested a constant temperature water bath at 37 °C for approximately 1 h. Finally, the capillaries were separated with Percoll and seeded in a 6-well plate containing a unique medium for pericytes. After 72 h, the medium was changed for the first time, and new medium was added every 2 days. PDGFR-β and CD31 antibodies were used for immunostaining.

### Aβ uptake

Primary cells were incubated with 500 nM human HyLyte Fluor™ 555-labeled Aβ1-40 (Anaspec, Fremont, CA) for 2 h in serum-free media as previously described [33]. Following treatment, the cells were rinsed twice with 1X PBS without calcium and magnesium. We observed the fluorescence associated with Aβ under a fluorescence microscope and superimposed the cells under white light to observe the uptake of Aβ. The cells on the slides were fixed and processed for imaging according to the abovementioned immunocytochemistry protocol. After incubation with the primary anti-CD36 antibody (1:200), the cells were stained with DAPI to observe the colocalization of CD36 and Aβ1-40.

### Construction of in vitro BBB model

After separate culture of the BBB model, bEnd.3 cells (1.0 × 105 cells/cm^2^) were placed on the front side of the transwell and then cultured. For the contact cocultivation model, we referred to a previous method [34, 35]. Transwell cell membranes (24 wells, 0.4 μm) were seeded with pericytes (1.0 × 10^4^ cells/cm^2^) on the reverse side. After 6 h, the pericytes were firmly attached, bEnd.3 cells (1.0 × 10^5^ cells/cm^2^) were seeded on the front side of the transwell cell membrane and cultured. Pericytes and bEnd.3 cells were in contact through the small holes between the membranes. Generally, the fluid was changed every 2 days, and the BBB model was established for use in approximately 6 days.

### Transendothelial electrical resistance (TEER) determination

The TEER was measured with an EVOM2 resistance meter (WPI Company) [36]. After calibration as needed, we placed the long end of the electrode in the culture medium outside the cell insert and the short end in the upper culture medium of the cell (taking care not to contact the cell with the wall of the culture plate). The dynamic TEER value was used as the criterion for forming tight cell junctions: Ω measurement = Ω actual measurement- Ω blank. The area was used to calibrate TEER: Ω/cm^2^ = Ω measurement/S, where S is the area of the bottom film of the board [37].

### BBB permeability test

Permeability tests with dextran or Aβ labeled with fluorescein isothiocyanate (FITC) [32, 38, 39] were performed using FITC-dextran (molecular weight 40 kDa) and FITC-Aβ1-40 to evaluate the permeability of the in vitro BBB to larger molecules. After the BBB model was established, 100 nM or 1 µM Aβ1-40 was added to the lower chamber of the transwell container in the experimental group, and then assay buffer containing 5 µM FITC-dextran or FITC-Aβ1-40 (1 µM) was added. We further cultivated the cells in the lower chamber of the BBB system. At 0, 30, 60, 90, 120, and 180 min after the addition of fluorescein, 50 μl of the medium was collected from the upper chamber and supplemented with an equal volume of medium. A fluorescence microplate reader was used to detect the fluorescence intensity of the sample (excitation wavelength 485 nm; emission wavelength 520 nm). According to the standard curve of FITC-dextran/Aβ, the relative fluorescence unit was converted to the value of ng/ml, and the background fluorescence and serial dilution were corrected during the experiment.

### Aβ1-40 preparation

Aβ1-40 was mixed to 1 mM with hexafluoroisopropanol. The sample was vortexed to dissolve the mixture, stored in a fume hood to air dry overnight, resuspended in DMSO, mixed with 1 mM liquor, and aggregated at 37 °C. This sample was stored at − 80 °C and diluted with culture medium to the required concentration for use.

### Gene silencing with lentivirus-mediated siRNA

LV-CD36-RNAi was obtained from GeneChem. According to the manufacturer’s protocols, the lentivirus and negative control sequences were transduced into pericytes. The virus was added according to the appropriate multiplicity of infection (MOI). Puromycin (2 µg/ml) was added after 72 h to select cells. After 1 week of screening, the cells were passaged at 1:2 in T25 bottles, and the medium was changed every 2 days. qRT–PCR and immunoblotting experiments were used to detect the silencing efficiency of siRNA, which was then used for the follow-up experiments.

### Analysis of reactive oxygen species (ROS) production

After cell treatment, a lipid ROS fluorescent probe (BODIPY 581/591, 50 µM) was added for 1 h or an intracellular Fe^2+^ probe (Mito-FerroGreen, 5 µM) working solution was added, and the cells were incubated at 37 °C. After culture for 30 min, the cells were washed 3 times with serum-free medium. Finally, the cells were evaluated using a multifunctional fluorescence microplate reader.

### ROS fluorescent probe

Dihydroethidium (DHE), a superoxide anion fluorescent probe, is bound to DNA by ROS oxide in living cells, and the fluorescence is red. The Reactive Oxygen Species Assay Kit (also called ROS Assay Kit) contains the fluorescent probe DCFH-DA. ROS can oxidize this probe into green-emitting DCF in cells. The probe loading steps were as follows: DHE and DCFH-DA were diluted with serum-free medium to 5 µM and 10 µM, respectively. The cell supernatant was aspirated, the cells were washed twice with PBS, and DHE or DCFH-DA was added to cover the cells. Then, the cells were placed in a 37 °C cell incubator and incubated for 60 min. The cells were washed twice with PBS before observation, and images were obtained with a fluorescence microscope to remove the probes in the supernatant.

### Detection of mitochondrial ROS (MitoSOX) and mitochondrial membrane potential (Δψ)

The Δψ and MitoSOX assays were performed according to the manufacturer’s specifications. The cells were treated as described above. After stimulation with Aβ1-40, the cells were separated using trypsin and centrifuged at 3000 rpm for 5 min. Then, the cells were resuspended in pericyte culture medium and stained as follows. Mitochondrial ROS were incubated with 5 µM MitoSOX at 37 °C for 15 min, and mitochondrial membrane potential was determined with 100 nM TMRM at 37 °C for 30 min. After washing, the cells were subsequently measured using an LSR II flow cytometer (BD Biosciences) and analyzed using FlowJo software.

### Annexin V-FITC/PI double-label staining to detect cell apoptosis

1. The culture medium was discarded, and the cells were digested with 0.25% trypsin without EDTA at room temperature. After the cells were round and floating under the microscope, the digestion was immediately stopped with complete culture medium, and the cell suspension was transferred to a flow tube.
2. The sample was centrifuged at 1000 rpm, and the precipitate was left after 5 min.
3. The cells were washed twice with precooled PBS and resuspended at 1 × 10^6^ cells/ml in 1 × buffer.
4. One hundred microliters of the resuspended cell solution was placed in a flow tube.
5. Then, 5 µl of FITC Annexin V and 5 µl of PI were added.
6. The sample was vortexed gently and incubated for 5 min at room temperature in the dark.
7. Then, 400 µl of 1 × buffer was added to each tube for testing within 1 h. The cells were subsequently measured using an LSR II flow cytometer (BD Biosciences) and analyzed using FlowJo software.

The Annexin V-FITC/PI double labeling results were analyzed using FlowJo software, and the cells were categorized in the following four quadrants: lower left, live cells; lower right, early apoptosis; upper right, late apoptosis; and upper left, cell fragments. Data collection and analysis were performed.

### Measurement of adenosine-5ʹ-triphosphate (ATP) levels

ATP levels were determined using an ATP Bioluminescence Assay Kit following the manufacturer’s instructions. Briefly, the supernatant was discarded, lysis solution was added, and the samples were shaken to fully lyse the cells (0.2 ml was added to each well of the 6-well plate). After lysis, the cells were transferred to a 1.5 ml EP tube and placed in a 4 °C centrifuge at 12,000 rpm for 10 min. After washing, the protein concentration was determined by the BCA method. First, 100 µl of ATP detection working solution was added to each well of a black 96-well plate. Then, 20 µl of sample or standard (drawn ATP standard curve) was added and mixed quickly. The samples were immediately tested in multifunctional microplate reader (luminometer) mode. The ATP concentration was converted to µmol/µg according to the protein concentration.

### Detection of superoxide dismutase (SOD) in the cell supernatant

The protocol was performed according to the SOD detection kit manual (Nanjing Jiancheng Bioengineering Institute, A001-3). First, after the cells were treated according to the above method, the cell supernatant was transferred to a 1.5 ml EP tube and centrifuged at 2000 rpm for 5 min. After the cells were lysed, the protein concentration was determined by the BCA method, and the samples were used for subsequent experiments. After thorough mixing, the mixture was incubated in a 37 °C incubator for 20 min and measured at 450 nm with a microplate reader. Finally, the OD value was converted to U/ml.

### Glutathione peroxidase (GSH-PX) detection

With a GSH-PX test kit (Nanjing Jiancheng Bioengineering Institute, A005), the enzyme activity can be calculated according to the consumption of reduced glutathione in the enzymatic reaction. Cell protein was extracted according to the above method, and BCA assays were used to measure the protein concentration of GSH-PX. First, the optimal sampling concentration was determined, and an inhibition rate of 45% to 50% was taken as the optimal sampling concentration. The inhibition rate formula was as follows: inhibition rate = (nonenzyme tube-enzyme tube) OD value/nonenzyme tube-enzyme tube OD value × 100%, and the result was between 15 and 55%. Then, the OD value of each tube was measured at a wavelength of 412 nm according to the instructions, and the GSH-PX activity was calculated.

### Iron content detection

We used an iron colorimetry kit (Elabscience Biotechnology Co., Ltd.); ferrous iron and pyridine combine to form a pink complex, and the amount of iron ions is proportional to the color. According to the above method, the cell protein was extracted, and BCA was used to measure the protein concentration for quantification of iron ion levels. The protocol was as follows. (1) The blank tube contained 500 µl of deionized water; the standard tube had 500 µl of standard application solution at a concentration of 2 mg/l, and the measurement tube had 500 µl of the sample to be tested. The samples were separately added to 5 ml EP tubes. (2) Then, 1.5 ml of iron developer was added to each tube (1), and the tubes were vortexed, mixed well, and boiled in boiling water at 100 °C for 5 min. (3) The samples were cooled with running water and centrifuged for 10 min at 2300×*g*. (4) Then, 250 µl of each supernatant was added to a 96-well plate, and the absorbance was measured at a wavelength of 520 nm. The iron content was calculated according to the following formula: iron content (mg/gprot) = (sample-blank) OD value/(standard-blank) OD value × standard concentration (2 mg/l) ÷ test sample protein concentration (gprot/l).

### CCK-8 assay of cell proliferation

This assay was conducted following the instructions of the Cell Counting Kit-8 (Dojindo, CK04). The steps were as follows. (1) First, 100 µl of cell suspension was prepared in a 96-well plate, and the culture plate was placed in an incubator for 24 h (at 37 °C, 5% CO_2_). (2) After Aβ1-40 treatment, the sample was added to the culture plate. (3) The culture plate was incubated in an incubator for 0, 6, 12, 18, and 24 h. (4) Then, 10 µl of CCK-8 solution was added to each well. (5) The culture plate was incubated in the incubator for 2 h. (6) The absorbance was measured at 450 nm with a microplate reader.

### Immunofluorescence and confocal imaging

After the various treatments, primary cultured mouse cells seeded on coverslips were fixed with 4% ice-cold paraformaldehyde at room temperature for 15 min followed by permeabilization with 0.1% Triton X-100/PBS. After incubation in blocking solution (2% BSA/PBS) at room temperature for 1 h, the cells were incubated with primary antibody at 4 °C overnight. This step was followed by three washes in PBS. The cells were then incubated with goat anti-rabbit IgG-Alexa 594 (Abcam), goat anti-rabbit IgG-Alexa 488 (Abcam), goat anti-mouse IgG-Alexa 594 (Abcam), goat anti-mouse IgG-Alexa 488 (Abcam), or goat anti-mouse IgG-Alexa 594 (Arigo) for 1 h at room temperature. After the cells were washed with PBS 3 times, the coverslips were mounted onto glass slides using Fluorescent Mounting Medium. For visualization of acidic lysosomal compartments, cells were stained with LysoTracker Red (Thermo Fisher Scientific) for 1 h according to the manufacturer’s instructions. After incubation with LysoTracker Red, cells were fixed in 4% PFA/PBS for 15 min at room temperature. The cells were incubated with primary antibody at 4 °C overnight. The cells were then washed in PBS 3 times followed by staining with 2 mg/ml Hoechst 33,342 for 5 min at room temperature. The cells were washed with PBS four times, and coverslips were mounted on glass slides using Fluorescent Mounting Medium. All confocal images were acquired and processed using a confocal microscope (Nikon).

### Western blotting

Proteins from animal brains were homogenized, and cells were lysed with RIPA buffer. The samples were loaded on SDS–PAGE (8–12% acrylamide) gels, and the total protein concentration was determined by BCA assays. The same amount of protein (20–40 mg) was loaded and run on SDS–PAGE gels, and the proteins were then transferred to nitrocellulose membranes (0.45 or 0.22 μm), which were blocked with 5% nonfat dry milk in 0.01 M PBS (pH 7.4) and 0.05% Tween-20 (PBST) at room temperature for 1 h. Subsequently, the membrane was incubated with primary antibodies directed against target proteins overnight at 4 °C. The final dilutions for primary antibodies were as follows: after three quick washes in PBST, the membranes were incubated with secondary antibodies conjugated to horseradish peroxidase (Abcam) diluted 1:3,000 in PBST for 1 h. The final detection of immunoreactive bands was developed using an enhanced chemiluminescent Western blot system (ECL) with exposure to a chemiluminescence imaging system (UVP). The immunoblotting signal intensity was measured using ImageJ 64 software.

### Electron microscopy

For scanning electron microscopy, the cells were grown and processed as described above. First, the cells were fixed with 2.5% glutaraldehyde at 4 °C overnight and then washed three times with PBS. After being fixed with 1% osmium acid for 1.5 h at 4 °C, the samples were dehydrated in a graded ethanol series and embedded in acrylic resin at 60 °C for 48 h. Then, 70-nm ultrathin sections were mounted on a nickel grid. The samples were stained with uranyl acetate for 10–20 min and lead citrate for 5–10 min and rinsed thoroughly with distilled water. A 120 kV Hitachi transmission electron microscope was used to capture images of the sample.

### Quantitative real-time PCR and sequencing

Total RNA was extracted from cells using TRIzol (Invitrogen) and further washed using RNase-free water. RNA was reverse-transcribed with a PrimeScript RT Reagent kit (TaKaRa) for cDNA synthesis, and the cDNA was then amplified by real-time PCR with a SYBR Premix ExTaq kit (TaKaRa). The relative expression of genes was normalized to the expression of the housekeeping gene GAPDH. The primer sequences were as follows: GAPDH forward, 5′-GTTCCTACCCCCAA TGTGTCC-3′ and reverse, 5′-TAGCCCAAGA TGCCCTTCAGT-3′; CD36 forward, 5′- ACCTTTTGTTGAGAAGTCTCGAAC-3′ and reverse, 5′- CTTTTTCAGTGCAGAAACAGTGG-3′; LRP1 forward, 5′- CTGGTCGATAGCAAGATTGTATTTC-3′ and reverse, 5′- GTGGCGTAGAGGTAGTTCTCAAAC -3′; HSP60 forward, 5′- CAGTCCTTCGCCAGATGAGAC -3′ and reverse, 5′- GGGACTTCCCCAACTCTGTTC -3′; and TIM23 forward, 5′- ACTGGTATGAACCCCCTGTCTC -3′ and reverse: 5′- CTGAGTTTCCTTCAATCCTAAACG -3′. Analysis was performed with three biological replicates and three independent technical replicates for each sample, with the average value taken and normalized to GAPDH gene levels to give the threshold cycle (ΔCT) values.

### Statistical analysis

All data are expressed as the mean ± standard deviation, and SPSS 23.0 software (SPSS, Inc., Chicago, IL, USA) was used for the analysis of significant differences. GraphPad Prism 8.0 software was used for graphing. T tests were used for comparisons between two groups, one-way analysis of variance was used to compare multiple groups, and Student-Newman–Keuls (SNK) tests were used for comparisons between groups. For repeated measurement data, repeated measurement analysis of variance was used. For the comparison of data between two groups, Student’s t test was used when the variance was uniform, and Welch’s t test was used when it was not uniform. P < 0.05 was considered to indicate a significant difference.

## Results

### The BBB of APP/PS1 mice is disrupted, with the number of pericytes decreased, and CD36 may be involved in Aβ uptake

**Fig. 1:**
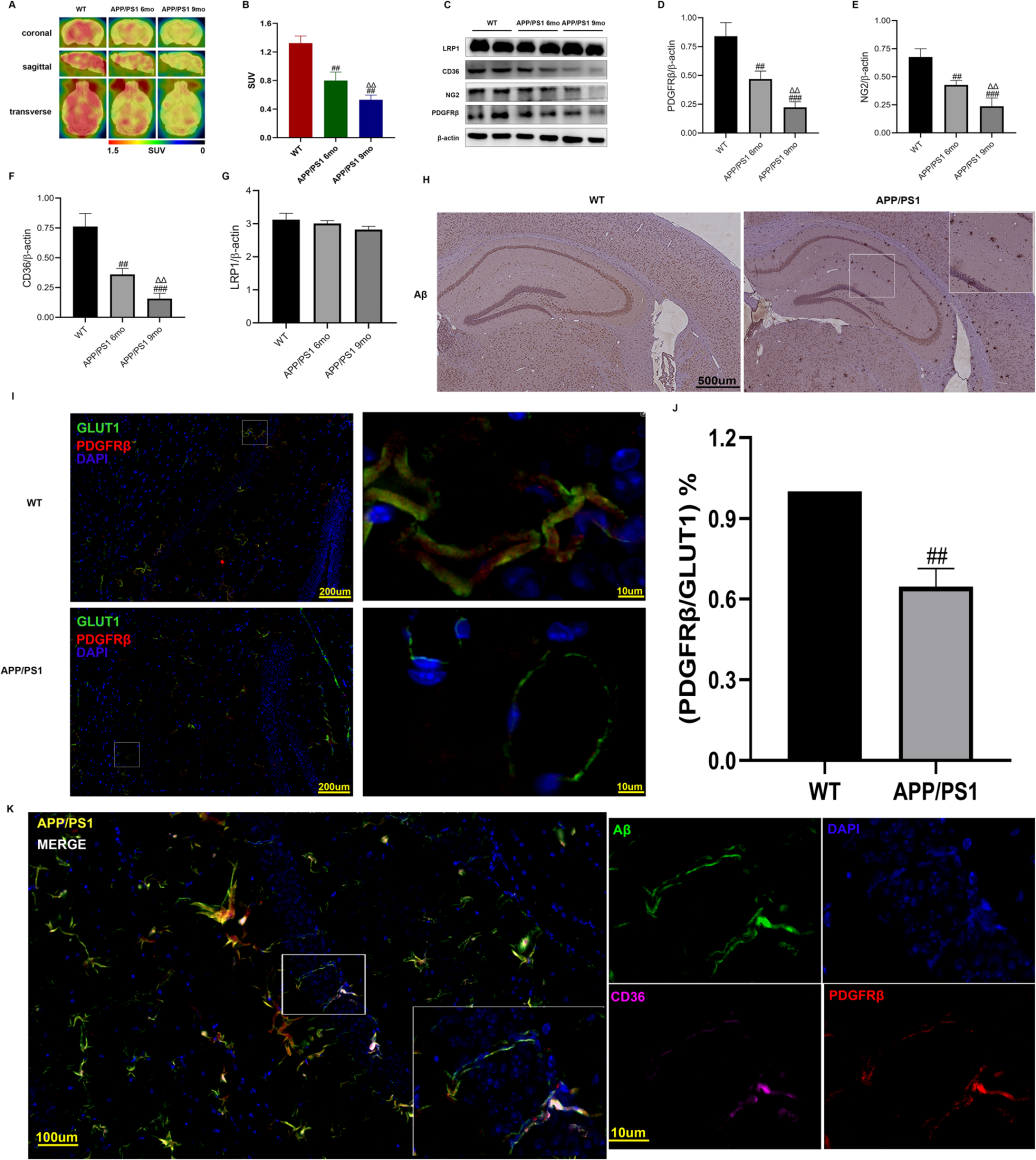
The APP/PS1 mouse BBB is damaged, pericytes are reduced, and CD36 expression is reduced. Pericytes express CD36 and colocalize with Aβ1. **A** PET/CT scan images of mice in each group. Brain glucose metabolic images of the APP/PS1 mice and the WT mice (taken 0–1 min after 18F-FDG injection). **B** The mean standardized uptake value (SUV) (n = 3). **C** Protein expression levels of PDGFRβ, NG2, CD36, and LRP1 in brain tissue. **D**–**G** Semiquantitative analysis of PDGFRβ, NG2, CD36, and LRP1 protein levels. The data are presented as the means ± SD. **H** Immunohistochemical (IHC) staining shows the formation of amyloid plaques composed of Aβ. **I** Immunofluorescence showed that there were fewer APP/PS1 mouse endothelial cell (GLUT1 pink label)-covered pericytes (PDGFRβ green label) than those of the WT mice. **J** Fluorescence semiquantitative analysis of pericytes and endothelial cells. We randomly selected six 200 µm visual fields for statistical analysis (6 areas of the cortex and hippocampus were used for image acquisition). **K** Immunofluorescence shows that CD36 (pink) and Aβ (green) are colocalized in pericytes (red). Blue indicates DAPI. (^##^p < 0.01, ^###^p < 0.001 compared with the WT group, ^ΔΔ^p < 0.01 compared with the APP/PS1 6 mo group, n = 6)

Normal glucose metabolism in brain tissue requires an intact BBB. Under normal circumstances, glucose uptake by brain tissue is maintained at a high level. As neuronal apoptosis and disruption of the BBB increase in APP/PS1 mice, glucose uptake in the brain tissue begins to decrease. We first observed 18F-FDG uptake in brain tissue with PET/CT. Blood perfusion imaging of 9-month-old and 6-month-old APP/PS1 mice showed an asymmetrical reduction in frontal and temporal lobe perfusion, and we found that 18F-FDG uptake was reduced (Fig. 1A, B).

The immunoblotting results of the total proteins in the cerebral cortex showed that the expression levels of the pericyte-specific proteins PDGFRβ and NG2 in the APP/PS1 mice gradually decreased. The levels were significantly different compared with those in the WT group. CD36 expression was most significantly lower in the 9-month-old APP/PS1 mice compared with WT mice. In contrast, the expression level of the Aβ transporter LRP1 was not substantially different from that in the WT group (Fig. 1B–G).

Previous studies have confirmed that the lack of CD36 can offset cerebrovascular dysfunction in Tg2576 mice and partially normalize the number and morphology of pericytes [29]. In this study, we confirmed by immunohistochemistry that 6-month-old APP/PS1 mice had Aβ plaques (Fig. 1H), and the pericyte numbers decreased (Fig. 1I, J). To determine whether pericytes uptake Aβ through CD36, we performed immunofluorescence co-staining for CD36, Aβ, and PDGFRβ (labeled pericytes) on APP/PS1 mouse brain slices to detect whether the three proteins colocalized. Our results (Fig. 1K) suggested that CD36 might be involved in Aβ uptake of pericytes (Additional file 1: Figure S1). However, the specific mechanism that affects the BBB is unknown. Therefore, we designed an in vitro BBB model to further verify the role of pericyte CD36 in the BBB.

### Pericytes uptake Aβ1-40 via CD36, causing injury to the BBB in vitro

#### CD36 is involved in the uptake of Aβ1-40 by pericytes

**Fig. 2:**
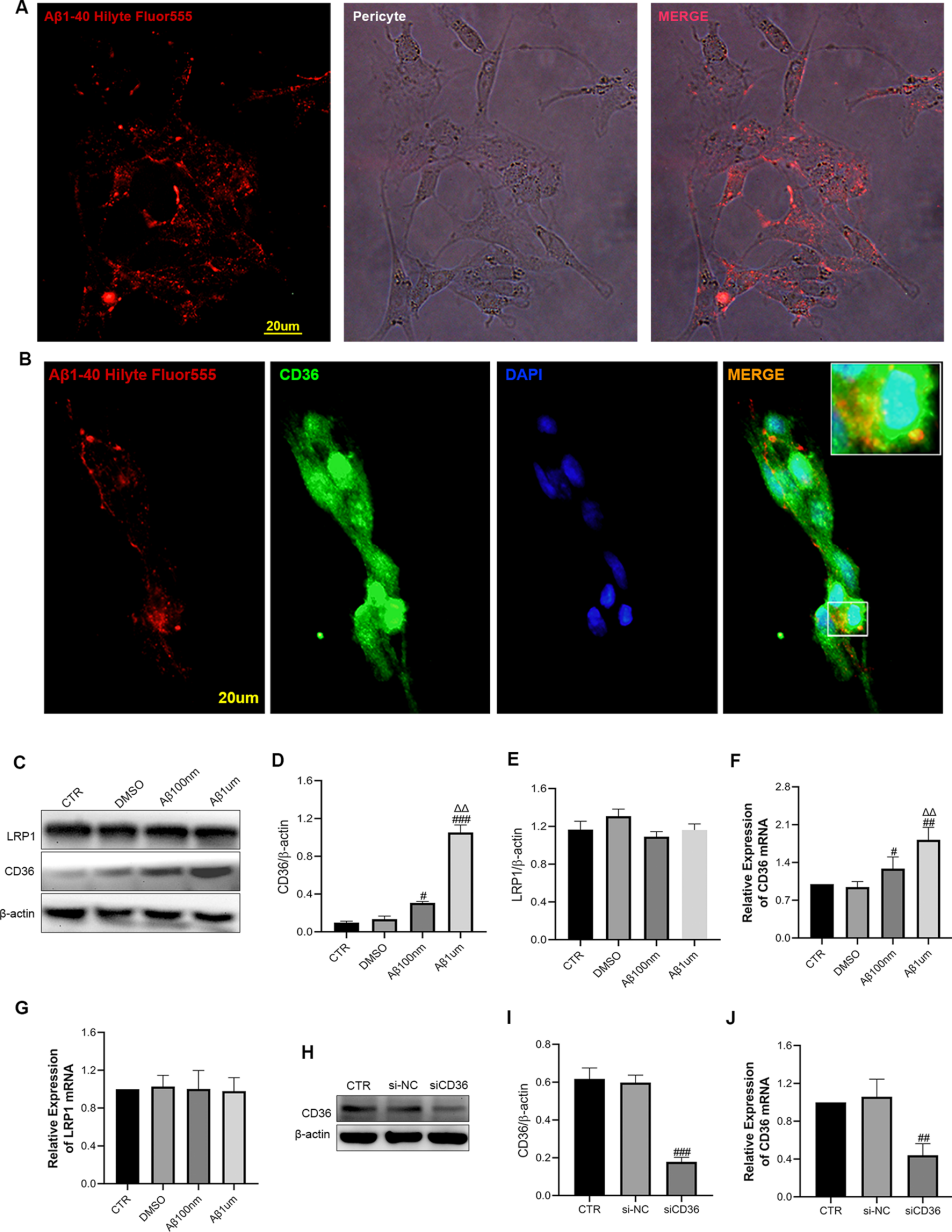
Aβ1-40 is transported to pericytes via CD36 and upregulates the expression of CD36 protein. **A** Aβ1-40 was incubated with pericytes for 1 h, and the pericytes overlapped with red fluorescent Aβ1-40 under an optical microscope. **B** Cellular immunofluorescence showed red fluorescent Aβ1-40 in the pericytes and CD36 (green light). Colocalization was observed. Blue indicates DAPI, scale 20 µm. **C** Western blotting was used to detect CD36 and LRP1 protein expression after 6 h of treatment of pericytes with Aβ1-40 (100 nM and 1 µM). **D**, **E** Semiquantitative analysis of CD36 and LRP1 protein levels. The data are presented as the means ± SD (n = 3 per group). **F**, **G** qRT–PCR was used to detect the changes in the transcriptional levels of CD36 and LRP1 in Aβ1-40 (100 nM and 1 µM)-treated pericytes for 6 h. **H** Western blots were used to detect CD36 expression at the protein level after RNA interference. **I** Semiquantitative analysis of CD36 protein levels. The data are presented as the means ± SD (n = 3 per group). **J** qRT–PCR detected the expression changes induced by si-CD36 at the transcriptional level (^#^p < 0.05, ^##^p < 0.01, ^###^p < 0.001 compared with the CTR group, ^ΔΔ^p < 0.01 compared with the 100 nM Aβ1-40 group, n = 3). si-NC indicates the control group

To verify whether the transport of Aβ1-40 into cells involves CD36, we used HiLyte Fluor™ 555-Aβ1-40 with red light. Then, immunofluorescence staining for CD36 and light microscopy revealed pericytes under white light, and the red fluorescence of Aβ1-40 overlapped with the pericytes (Fig. 2A), suggesting that Aβ1-40 was transported into pericytes. Furthermore, we found that CD36 (green) immunofluorescence and Aβ1-40 (red) fluorescence colocalized in pericytes (Fig. 2B), confirming that CD36 was involved in the uptake of Aβ1-40 by pericytes.

#### A low dose of Aβ1-40 leads to upregulation of CD36 expression in pericytes, while the transporter LRP1 is not affected

To verify the effect of Aβ1-40 on the expression of pericyte transporters, we used two doses of Aβ1-40 (100 nM and 1 µM) to investigate the cell level. After treatment with 100 nM and 1 µM Aβ1-40 and incubation with pericytes for 6 h, both the protein level (Western blot) (Fig. 2C, D) and the transcription level (PCR) (Fig. 2F) indicated that CD36 expression was significantly increased after stimulation with Aβ1-40. In addition, the concentration of 1 µM Aβ1-40 induced a significantly higher increase (p < 0.001). At the same time, the protein level (Fig. 2C, E) and the transcription level (Fig. 2G) of the amyloid efflux transporter LRP1 did not change significantly. To verify the function of CD36 in the BBB, we performed RNA interference to downregulate CD36 expression at the gene level and transferred the si-CD36 plasmid via lentivirus to alter expression at the protein level (Fig. 2H, I) and transcription level (Fig. 2J). We found that the CD36 expression level after RNA interference was significantly lower than that in the control group, confirming the efficacy of the interference. Moreover, there was no difference between the control group and the empty vector control group (si-NC group). After downregulating the expression of CD36, we performed follow-up studies of the BBB in vitro.

#### Aβ1-40 increases the permeability of the BBB, and downregulating CD36 expression in pericytes improves the permeability changes in the BBB induced by Aβ1-40

Aβ1-40 is easily deposited in the vascular system [21], its impact on microvessels lies in the influence on endothelial cells and pericytes which share the basement membrane with each other. Pericytes also help regulating capillary diameter and cerebral blood flow which lead to changes in the BBB functions. The choice of clinical treatment targets on BBB is pivotal. To verify the effect of Aβ1-40 on the permeability of the BBB, we cultured bEnd.3 cells separately and cocultured primary pericytes and bEnd.3 cells in an in vitro transwell system to establish a tight junction-type in vitro BBB model.

**Fig. 3:**
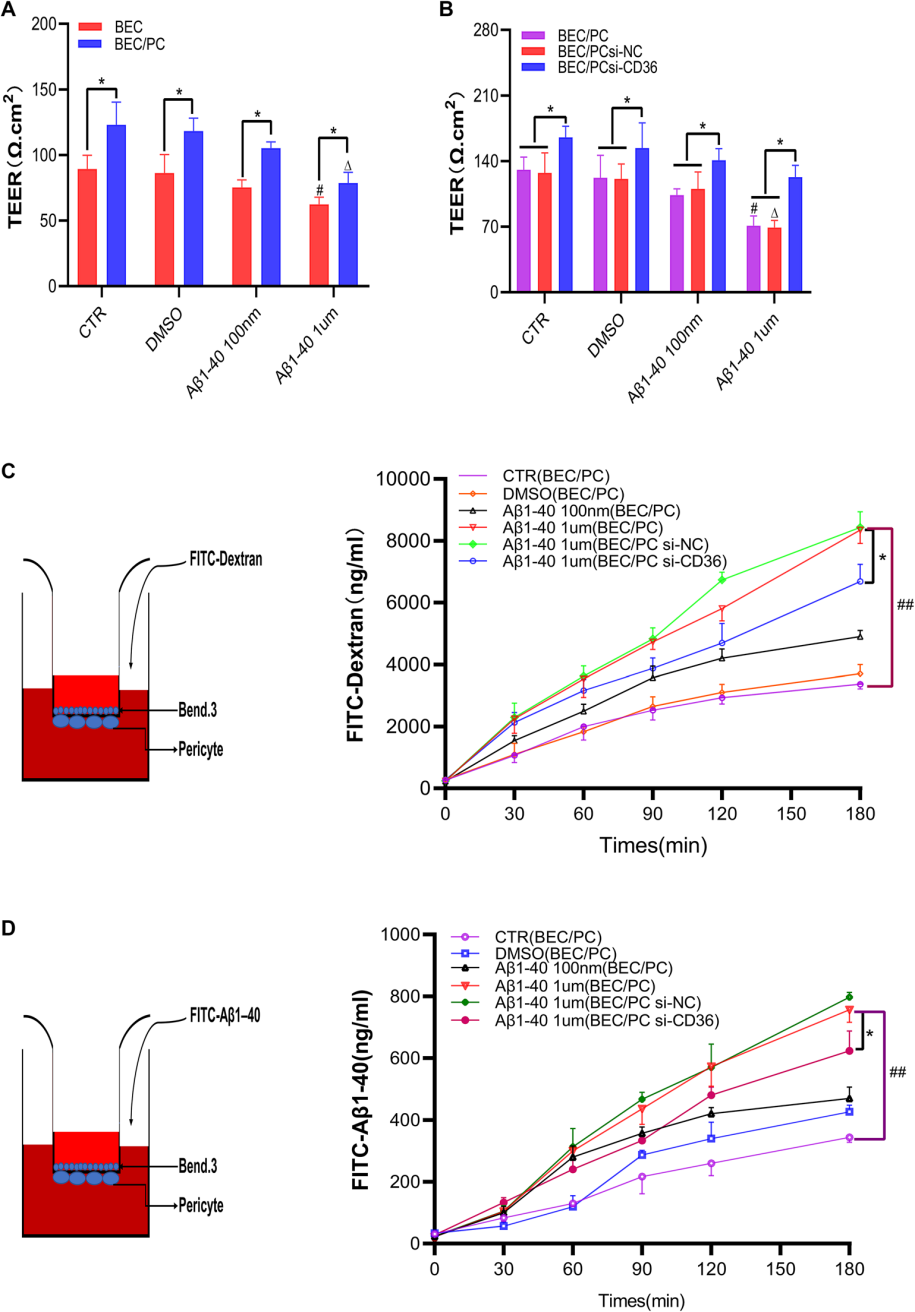
Aβ1-40 increases the permeability of the BBB, and downregulating the expression of CD36 can improve the BBB changes caused by Aβ1-40. **A** TEER detects the BBB tightness of Aβ1-40-treated cells (100 nM and 1 µM) in monolayer and coculture models for 6 h. **B** TEER detects the tightness of the BBB between the Aβ1-40 (100 nM and 1 µM)-treated pericyte si-CD36 coculture group and the control group after 6 h [*p < 0.05 in A and B compared with the same group, #p < 0.05 compared with the BEC single-layer model CTR group, Δp < 0.05 compared with the BEC/PC (**A**) or BEC/PC si-NC (**B**) coculture model CTR group, n = 3]. **C** The fluorescence microplate reader detects the normalized permeability of FITC-dextran after 1 µM Aβ1-40 treatment of the coculture model. **D** The fluorescence microplate reader detects the normalized permeability of FITC-Aβ1-40 after 1 µM Aβ1-40 treatment of the coculture model. (^##^p < 0.01 vs. the BEC/PC group, *p < 0.05 vs. the BEC/PC si-NC group, n = 3). *BEC* bEnd.3 is cultured alone, *BEC/PC* bEnd.3/pericyte coculture, *si-NC* empty vector control

To investigate the effect of CD36 pericytes on the permeability of the BBB, we inhibited CD36 expression by lentiviral transfection through RNA interference and then cocultured primary pericytes and bEnd.3 cells in the transwell system to establish a contact-type in vitro BBB model. Endothelial cells were seeded on the front side of the transwell cell membrane, and pericytes were seeded on the back side of the cell membrane. The two cell types were in contact through the intermembrane pores (pore size, 0.4 µm). This model could ideally simulate the in vivo BBB structure (Fig. 3C, simulation diagram in D).

In this experiment, after construction of an in vitro BBB model, Aβ1-40 (100 nM and 1 µM) was first added to the lower chamber of a transwell plate in a single culture and coculture model for 6 h. Then, the medium was changed, and the connectivity of the BBB was tested using TEER; the change reflects the influence of Aβ1-40 on the BBB. We found that the TEER in the coculture model was significantly higher than that in the bEnd.3 single-culture models (Fig. 3A). The difference was significant. Aβ1-40 (1 µM) dramatically reduced the TEER value of BBB cells cultured alone and cocultured (Fig. 3A). The difference was significant (p < 0.05), while 100 nM Aβ1-40 had no effect on tight junctions in the BBB cells cultured alone and cocultured (Fig. 3A). These results indicated that pericytes could increase the tightness of the BBB and that Aβ1-40 damaged the BBB in a dose-dependent manner.

After inhibition of CD36 expression in pericytes, the TEER value in the BEC/PC si-CD36 group in the cocultured transwell model was significantly higher than that in the control group (p < 0.05) (Fig. 3B). However, the TEER value in the 1 µM Aβ1-40-treated group was significantly decreased compared with that in the control group 6 h after Aβ1-40 was added to the lower chamber, while the value in the BEC/PC si-CD36 group was also reduced. Nevertheless, there was no significant difference compared with the control group. Permeability testing of FITC-labeled Aβ1-40 and dextran (dextran, 40 kDa) was performed; 1 µM Aβ1-40 was added to the lower chamber 6 h after the BBB permeability increased significantly, and after inhibiting CD36, the labeled Aβ1-40 and the penetration of dextran in the BEC/PC si-CD36 group were markedly lower than those in the BEC/PC group (Fig. 3C, D). The above results indicated that 1 µM Aβ1-40 increased the permeability of the BBB, and inhibiting CD36 expression in pericytes could reduce the permeability of the BBB and increase its compactness.

#### A low dose of Aβ1-40 inhibits pericyte proliferation, causes mitochondrial damage, and promotes mitophagy

**Fig. 4:**
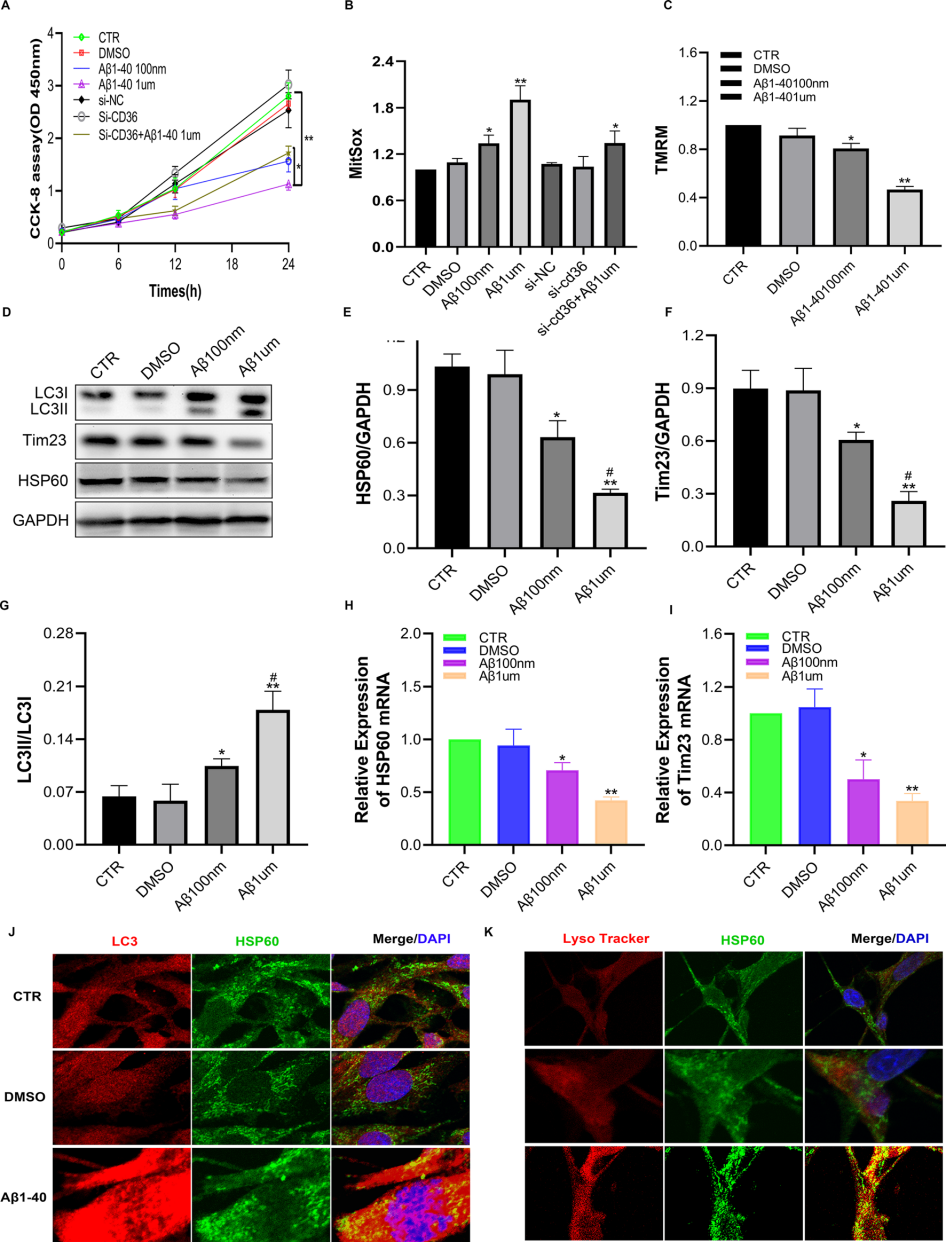
Mitochondrial autophagic changes after 6 h of treatment of pericytes with low-dose Aβ1-40 (100 nM and 1 µM). **A** The CCK-8 assay was used to detect the proliferation of pericytes after stimulation with Aβ1-40. **B**, **C** Flow cytometry was used to detect the effect of Aβ1-40 on mitochondrial damage after pericytes (MitoSOX: mitochondrial ROS, TMRM: mitochondrial membrane potential). **D** Western blotting was used to detect the expression changes of Tim23, HSP60, and LC3II/LC3I at the protein level. **E**–**G** Semiquantitative analysis of Tim23, HSP60, and LC3II/LC3I protein levels. The data are presented as the means ± SD (n = 3 per group). **H**, **I** qRT–PCR was used to detect the expression changes of HSP60 and Tim23 at the transcriptional level. **J** Immunofluorescence was used to detect the colocalization of autophagosomes (LC3 red) and mitochondria (HSP60 green). **K** Confocal microscopy with a lysosomal tracer (LysoTracker Red) and mitochondrial marker (HSP60 green) was used to colocalize proteins. (*p < 0.05, **p < 0.01 compared with the CTR group, ^#^p < 0.05 compared with the 100 nM Aβ1-40 group, n = 3)

To study the effect and influence of low-dose Aβ1-40 on pericytes, we incubated pericytes with different concentrations (100 nM and 1 µM) of Aβ1-40 for 6 h to observe the impact on pericyte proliferation and mitochondria. In this study, a CCK-8 kit was used to detect the effect of Aβ1-40 on pericyte proliferation. The results showed that pericyte proliferation was significantly inhibited after Aβ1-40 treatment for 12 h (Fig. 4A), and 1 µM resulted in the maximum inhibition at 24 h. The results were significant (p < 0.01), indicating that a low dose of Aβ1-40 could inhibit pericyte proliferation in a time- and concentration-dependent manner.

We also used flow cytometry to observe the effects of Aβ1-40 stimulation on pericyte mitochondrial damage and found that Aβ caused a significant increase in mitochondrial ROS (MitoSOX) compared with that in the control group (Fig. 4B). In contrast, the mitochondrial membrane potential (TMRM) was significantly lower than that in the control group (Fig. 4C), with 1 µM showing the most significant effect (p < 0.01). This finding indicated that with an increasing Aβ1-40 concentration, the damage to pericyte mitochondria was more serious and concentration-dependent.

To explore the effect of Aβ1-40 treatment on mitochondria, we evaluated the mitochondrial matrix protein HSP60 (Fig. 4D, E) and the inner membrane protein Tim23 (Fig. 4D, F). The results showed that the expression of these two mitochondrial proteins was significantly reduced after Aβ1-40 treatment (p < 0.01), and the expression of LC3II/LC3I (Fig. 4D, G) showed a significant increase (p < 0.01) after autophagic activation. These data indicate that Aβ1-40 treatment of pericytes caused an increase in mitochondrial autophagy in pericytes and promoted autophagy and the formation of autophagic bodies. We found that 1 µM Aβ1-40 showed a greater promotion of mitochondrial autophagy in pericytes than 100 nM Aβ1-40, and the difference was significant (p < 0.05). We also examined the expression of the mitochondrial matrix protein HSP60 (Fig. 4H) and the inner membrane protein Tim23 (Fig. 4I) at the RNA level after Aβ1-40 treatment. The results were consistent with the decrease in protein levels observed after Aβ1-40 treatment.

To verify the effect of Aβ1-40 on pericyte mitochondrial autophagy, we also used immunofluorescence to detect changes in autophagic flux. We examined the mitochondrial matrix protein HSP60 and autophagosomes (LC3) after treatment with 1 µM Aβ1-40 and observed colocalization of the proteins (Fig. 4J). To confirm that lysosomes indeed degraded mitochondria, we used a lysosomal tracer to co-stain mitochondria and lysosomes and found that 1 µM Aβ1-40 stimulated mitochondria and lysosomes. The colocalization of the lysosome (LysoTracker Red) was significantly increased (Fig. 4K), indicating that autophagic flux increased after stimulation with Aβ1-40; thus, Aβ1-40 induced lysosome-mediated mitochondrial degradation. However, there was no significant overlap between the control group and the DMSO group. Our results suggested that Aβ1-40 stimulated pericytes to increase mitochondrial autophagy, which involved mitochondria and fusion with lysosomes for degradation.

**Fig. 5:**
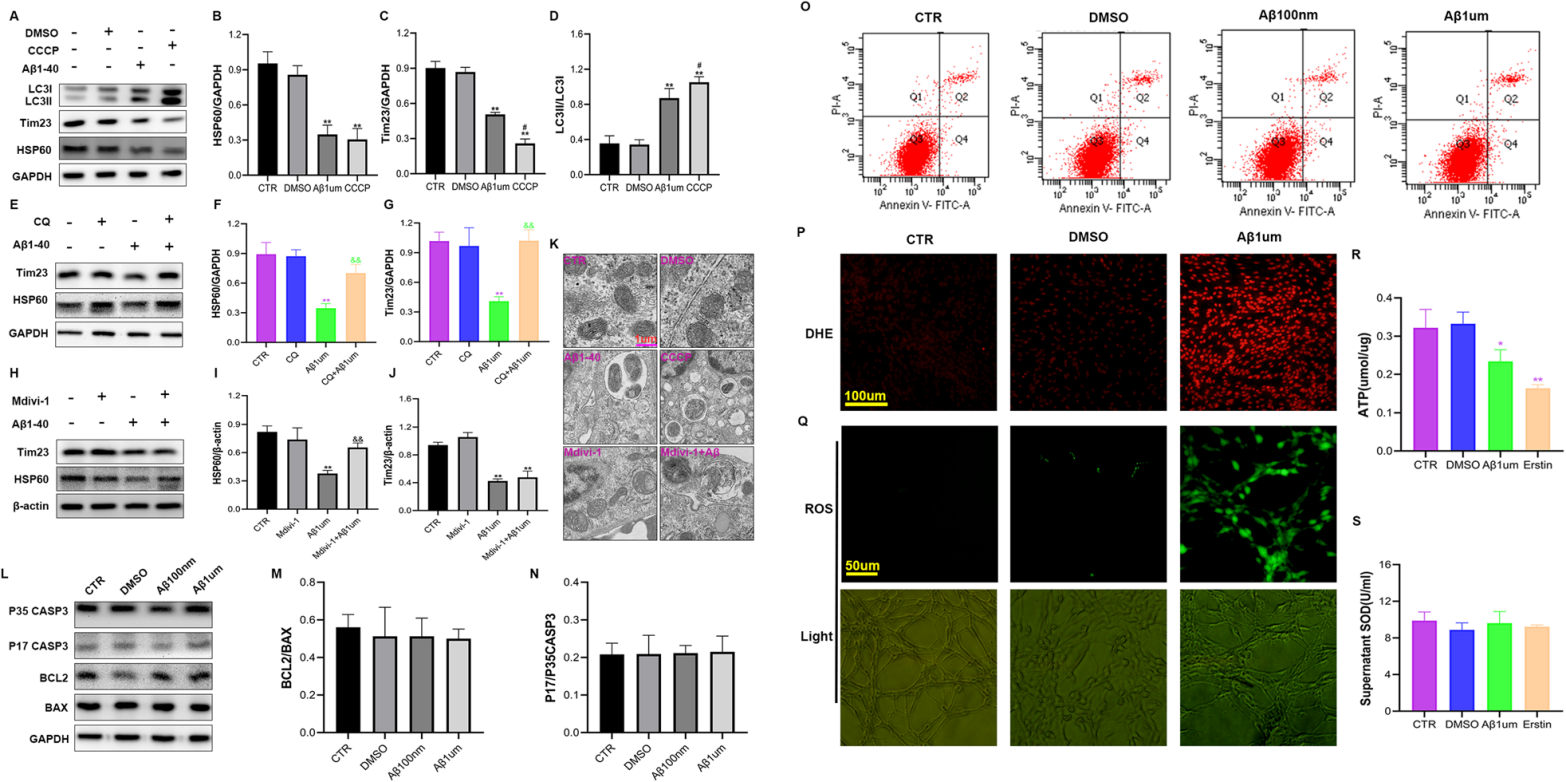
Autophagic agonists and inhibitors verified that 1 µM Aβ1-40 treatment of pericytes led to increased mitochondrial autophagy and increased the generation of ROS. **A** Western blot detection of the mitochondrial proteins Tim23 and HSP60 and the autophagosome protein LC3II/LC3I at the protein level after 5 µM CCCP treatment as a positive control. **B**–**D** Semiquantitative analysis of Tim23, HSP60, and LC3II/LC3I protein levels. The data are presented as the means ± SD (n = 3 per group). **E** Western blots to detect the expression changes in the mitochondrial proteins Tim23 and HSP60 at the protein level with or without 20 µM CQ. **F**, **G** Semiquantitative analysis of Tim23 and HSP60 protein levels. The data are presented as the means ± SD (n = 3 per group). (H) Western blot to detect the expression changes of mitochondrial proteins Tim23 and HSP60 at the protein level with or without Mdivi-1 20 µM. **I**, **J** Semiquantitative analysis of Tim23 and HSP60 protein levels. The data are presented as the means ± SD (n = 3 per group). (**p < 0.01 compared with the CTR group, ^&&^p < 0.01 compared with the 1 µM Aβ1-40 group, n = 3). **K** Use of 5 µM CCCP as a positive control and typical transmission electron micrographs after 6 h with or without 20 µM Mdivi-1. At least 6 cells were examined under each treatment condition. **L** Western blotting was used to detect changes in the expression of apoptosis-related BCL2/BAX and P17/P35 CASP3 after Aβ1-40 (100 nM and 1 µM) stimulation of pericytes for 6 h. **M**, **N** Semiquantitative analysis of BCL2/BAX and P17/P35 CASP3 protein levels. The data are presented as the means ± SD (n = 3 per group). **O** Flow cytometry to detect the effect of Aβ1-40 on pericyte apoptosis. **P** Fluorescence microscopy observation and detection of the fluorescent probe DHE indicate that Aβ1-40 stimulation changes ROS expression in pericytes. **Q** Fluorescence microscopy observation and detection of the fluorescent probe for ROS, which is used to show the changes in ROS expression in pericytes caused by Aβ1-40 stimulation. **R** The ATP kit detects the changes in pericyte energy produced by Aβ1-40 stimulation. **S** SOD kit detection of the effect of Aβ1-40 stimulation on the antioxidant capacity of pericytes (*p < 0.05, **p < 0.01 and CTR group, n = 3)

To further confirm that Aβ1-40 promoted pericyte mitochondrial autophagy, we applied the autophagic agonist carbonyl cyanide 3-chlorophenylhydrazone (CCCP, a respiratory chain uncoupler that reduces the mitochondrial membrane potential and induces mitochondrial autophagy; 5 μM) to treat pericytes for 6 h. The results showed that the levels of the mitochondrial matrix protein HSP60 (Fig. 5A, B) and the inner membrane protein Tim23 (Fig. 5A, C) were significantly lower than those of the control group. In contrast, the expression of LC3II/LC3I (Fig. 5A, D) after autophagic activation showed a significant increase, consistent with the results after exposure to 1 µM Aβ1-40. We also used the autophagy inhibitor chloroquine (CQ, which inhibits autophagosome and lysosome fusion by inhibiting autophagy; 20 µM) and added CQ 2 h before adding Aβ1-40. We found that after adding CQ, Aβ1-40 induced HSP60 (Fig. 5E, F) and Tim23 (Fig. 5E, G) expression. The decrease was reversed, and the levels of the two mitochondrial proteins were significantly higher than those in the Aβ1-40 group (p < 0.01). Electron microscopy data (5 K) showed that after pericytes were stimulated with Aβ1-40 or treated with CCCP, mitochondria were surrounded by autophagosomes/autolysosomes. The above results confirmed that Aβ1-40 induced an increase in mitochondrial autophagy in pericytes.

We also used the drug Mdivi-1 for verification. Mdivi-1 is an inhibitor of mitochondrial division that is often used to inhibit mitochondrial autophagy [40, 41]. After adding Mdivi-1 (20 µM) 2 h before adding Aβ1-40, we found that the reduction in HSP60 and Tim23 expression induced by Aβ1-40 was blocked by Mdivi-1 (Fig. 5H–J), and compared with the Aβ1-40 group, the treated group showed a significant difference (p < 0.01). After the treatment of pericytes with Mdivi-1, the electron microscopy results (Fig. 5K) showed that the area surrounding the pericyte mitochondria with autophagosomes/autolysates was better than that in the Aβ1-40 treatment group, and the number of mitochondria increased. The results showed that Mdivi-1 could inhibit mitochondrial autophagy caused by Aβ1-40. We verified that 1 µM Aβ1-40 induced an increase in mitochondrial autophagy in pericytes.

#### Low-dose Aβ1-40 does not cause pericyte cell apoptosis but increases oxidative stress

Mitochondrial damage and increased mitochondrial autophagy often lead to apoptosis. To determine whether Aβ1-40 (100 nM and 1 µM) caused pericyte apoptosis, we detected changes in apoptosis by flow cytometry and observed apoptosis at the protein level. We examined changes in proteins such as the antiapoptotic protein BCL2, the proapoptotic protein BAX, and the key apoptotic proteins caspase 3 (P35) and cleaved caspase 3 (P17). We found that BCL2/BAX (Fig. 5L, M) and P17/P35 CASP3 (Fig. 5L, N) levels did not change significantly after Aβ1-40 stimulation of pericytes compared with those in the control group. The apoptosis rate was detected by flow cytometry with Annexin-V/PI double staining to determine the changes in apoptosis; the results showed no difference (Fig. 5O). The above results indicate that a low dose of Aβ1-40 did not cause apoptosis within 6 h. However, pericyte mitochondria were damaged (Fig. 4), and the BBB was disrupted (Fig. 3). To further clarify the cause of cell damage, we detected oxidative stress.

ROS fluorescent probes (DHE and ROS kits) can freely penetrate the living cell membrane, can enter the cell and are oxidized by ROS in the cell to produce red and green fluorescence. Our study found that with 1 µM Aβ1-40, DHE red fluorescence (Fig. 5P) and ROS green fluorescence (Fig. 5Q) were significantly enhanced, leading to an increase in pericytic ROS, and the production of ATP (Fig. 5R) was considerably lower than that in the control group. During this process, SOD (Fig. 5S) showed no significant change. The above results indicate that Aβ1-40 led to an increase in cellular ROS production and a decrease in energy synthesis.

### A low dose of Aβ1-40 induces ferroptosis in pericytes

#### A low dose of Aβ1-40 induces an increase in iron ions and lipid ROS in pericytes, leading to increased ferroptosis

Ferroptosis is a recently identified form of cell necrosis. The primary mechanism is the accumulation of cellular lipid ROS in an iron-dependent manner [42]. The cystine/glutamate transporter SLC7A11 (also known as xCT) imports cysteine for glutathione biosynthesis and antioxidant defense [43]. Glutathione peroxidase 4 (GPX4) is an enzyme required to remove lipid ROS. Even under normal cellular cysteine and glutathione contents, this enzyme can induce iron-related death and is crucial for ferroptosis [44]. We found that stimulating cells with a low dose of Aβ1-40 increased ROS production in pericytes and damaged mitochondria. Therefore, we suspected that Aβ1-40 might cause ferroptosis in pericytes. To verify the above assumptions, we treated cells with 1 µM Aβ1-40 for one week. The cells were tested for related ferroptosis indicators after 6 h; erastin, an inducer of ferroptosis, was used as a positive control.

**Fig. 6:**
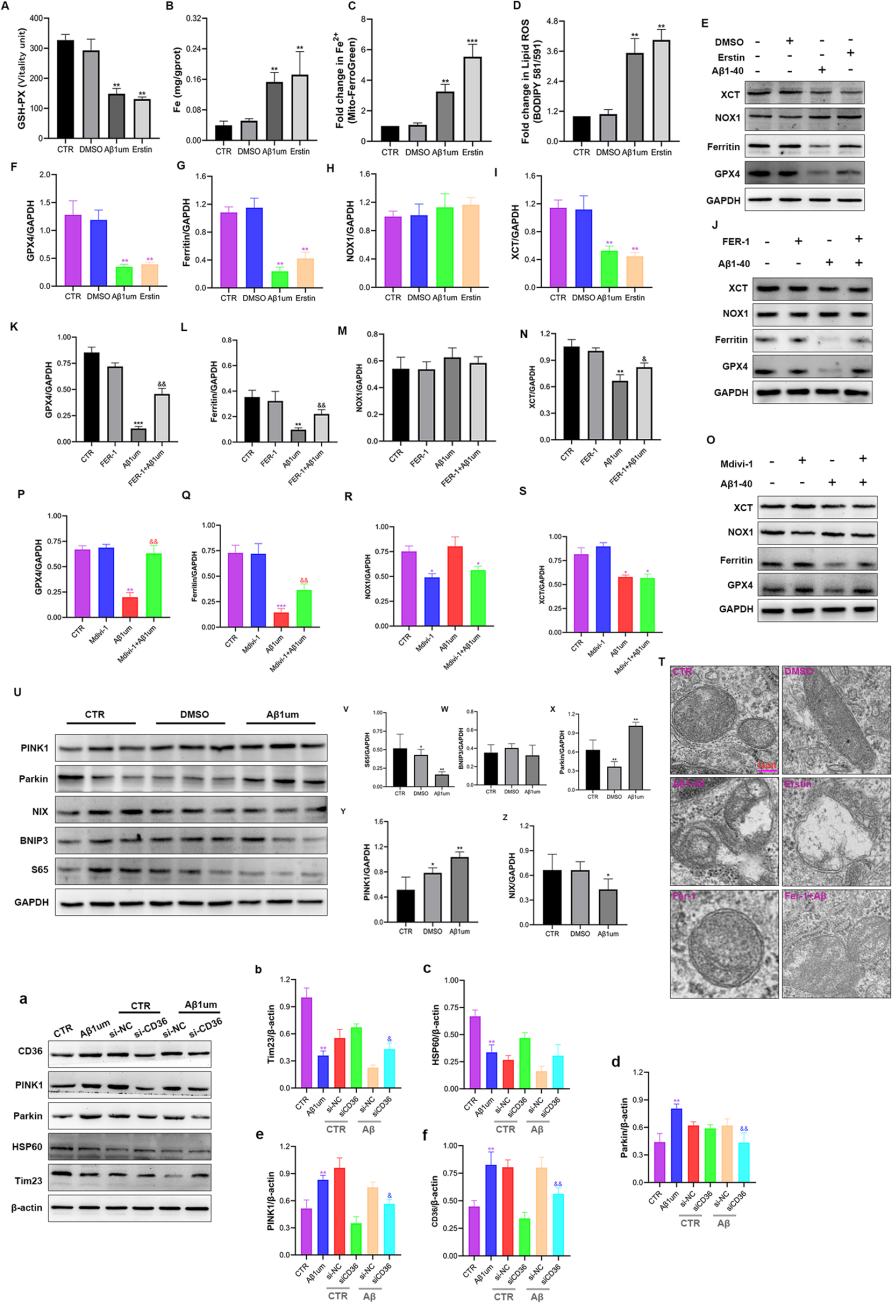
A low dose of Aβ1-40 causes cellular mitophagy-dependent lipid peroxidation and induces ferroptosis through CD36/PINK1/Parkin pathway. **A** The GSH-PX kit was used to detect the changes in pericyte glutathione peroxidase stimulated by Aβ1-40. **B** The iron kit detects changes in iron ions. **C** A fluorescence microplate reader (Mito-FerroGreen) was used to detect the change in Fe2 + . **D** Fluorescence microplate reader analysis of BODIPY581/591 detects the effect of lipid active oxygen. **E** Western blot analysis was used to detect changes in the expression of the ferroptosis-related proteins GPx4, xCT, ferritin, and NOX1 after stimulation of pericytes with 1 µM Aβ1-40 or 10 µM erastin for 6 h. **F–I** Semiquantitative analysis of GPx4, xCT, ferritin, and NOX1 protein levels. The data are presented as the means ± SD (n = 3 per group). **J** Western blotting was used to detect the changes in the expression of the ferroptosis-related proteins GPx4, xCT, ferritin, and NOX1 after pericytes were stimulated with 1 µM Aβ1-40 with or without FER-1 for 6 h. **K–N** Semiquantitative analysis of GPx4, xCT, ferritin, and NOX1 protein levels. The data are presented as the means ± SD (n = 3 per group). **T** Erastin (10 µM) was used as a positive control; typical transmission electron micrograph after 6 h with or without FER-1 (10 µM). At least 6 cells were examined under each treatment condition. **O** Erastin (10 µM) was used as a positive control; typical transmission electron micrograph after 6 h with or without FER-1 (10 µM). At least 6 cells were examined under each treatment condition. **P**–**S** Semiquantitative analysis of GPx4, xCT, ferritin, and NOX1 protein levels. The data are presented as the means ± SD (n = 3 per group). **U** Western blotting was used to detect the expression levels of the mitochondrial pathway proteins S65, BNIP3/NIX, and PINK1/Parkin after 1 µM Aβ1-40 treatment of pericytes for 6 h. **V**–**Z** Semiquantitative analysis of S65, BNIP3/NIX, and PINK1/Parkin protein levels. The data are presented as the means ± SD (n = 6 per group). **a** Western blot detection of the effect of 1 µM Aβ1-40 on the expression of CD36, PINK1/Parkin, HSP60, and Tim23 in pericytes 6 h after si-CD36 transfection. **b**–**d** Semiquantitative analysis of CD36, PINK1/Parkin, HSP60, and Tim23 protein levels. The data are presented as the means ± SD (n = 3 per group). (*p < 0.05, **p < 0.01, ***p < 0.001 compared with the CTR group, ^&^p < 0.05, ^&&^p < 0.01 compared with the 1 µM Aβ1-40 group, n = 3 or 6)

We found that Aβ1-40 caused a significant decrease in GSH-Px expression compared with the control group (Fig. 6A), while the level of iron ions increased significantly compared with the control group (Fig. 6B); the trends were the same as those after erastin stimulation. The results showed that both erastin and Aβ1-40 treatment of pericytes resulted in a significant increase in lipid ROS (Fig. 6C) and Fe2 + (Fig. 6D) compared with those in the control group. After the treatment of pericytes with erastin and Aβ1-40, the protein expression levels of GPx4, xCT, and ferritin (Fig. 6E–G, I) were significantly lower than those in the control group. In addition, NOX1 expression (Fig. 6E, H) was slightly increased compared with the control group; however, the difference was not significant. The above results show that Aβ1-40 induced ferroptosis of pericytes.

#### Low-dose Aβ1-40 causes ferroptosis, which is dependent on mitochondrial autophagy, in pericytes

We also applied ferrostatin-1 (FER-1), a ferroptosis inhibitor, to confirm that Aβ1-40 could induce ferroptosis in pericytes. Previous studies have shown that ferroptosis is an autophagic cell death process [45], thus, we used Mdivi-1 for verification. Through Western blotting, we found that 10 µM FER-1 blocked the reduction in the ferroptosis-related proteins GPx4, ferritin, and xCT induced by Aβ1-40 (Fig. 6J-L, N). The difference was significant compared with the Aβ1-40 group (p < 0.05 or 0.01). After the treatment of pericytes with Aβ1-40 or erastin, transmission electron microscopy (Fig. 6T) showed mitochondrial atrophy, increased membrane density, and necrosis-related vacuoles, which are morphological features of ferroptosis [46], and FER-1 reversed the mitochondrial changes associated with ferroptosis. The above results indicate that FER-1 could inhibit mitochondrial autophagy caused by Aβ1-40.

The results showed that after addition of Mdivi-1, the reduction in GPx4 and ferritin levels induced by Aβ1-40 could be reversed by Mdivi-1 (Fig. 6O–R), and the difference was significant compared with the Aβ1-40 group (p < 0.01). However, xCT did not change significantly after Mdivi-1 treatment of pericytes compared with that in the Aβ1-40 group (Fig. 6O, S). These results showed that Mdivi-1 could prevent ferroptosis by inhibiting cell mitochondrial autophagy and then upregulating the expression of GPx4. The above results indicated that the ferroptosis induced by Aβ1-40 was mediated by mitochondrial autophagy.

#### Low-dose Aβ1-40 induces mitochondrial autophagy in pericytes through the CD36/PINK1/Parkin pathway

To further explore the mechanism by which Aβ1-40 induced mitochondrial autophagy, we first detected the levels of the mitochondrial pathway proteins S65, BNIP3/NIX, and PINK1/Parkin in pericytes by Western blotting. We found that only PINK1/Parkin pathway proteins increased significantly after 1 µM Aβ1-40 treatment of pericytes (Fig. 6U, X, Y). Compared with the control group, the difference was significant (p < 0.01). S65 and BNIP3/NIX with 1 µM Aβ1-40 treatment decreased the protein level of pericytes (Fig. 6U–W, Z). The results showed that Aβ1-40 induced pericytes to activate mitochondrial autophagy through the PINK1/Parkin pathway.

To study the effect of CD36 on changes in the mitochondrial autophagic pathway, we downregulated the expression of CD36 in pericytes and then treated the cells with 1 µM Aβ1-40. The results showed that CD36 expression after si-CD36 treatment was significantly lower than that in the control group (Fig. 6a, f). Western blot results showed that si-CD36 blocked the expression of the pericyte mitochondrial pathway protein PINK1/Parkin (Fig. 6a, d, e) induced by 1 µM Aβ1-40, indicating that si-CD36 blocked PINK1/Parkin pathway activation caused by Aβ1-40. Moreover, si-CD36 reversed the downregulation of the Aβ1-40-induced mitochondrial protein HSP60 and Tim23 levels (Fig. 6a–c), indicating that si-CD36 could inhibit Aβ1-40-induced mitochondrial autophagy. The above results suggested that the promotion of mitochondrial autophagy by Aβ1-40 was mediated through the CD36/PINK1/Parkin pathway.

## Discussion

Aβ plays an essential role in AD [47]. The lack of CD36 in Tg2576 mice aged 3–4 months did not change Aβ levels in the brain [48]. Thus, in this study, APP/PS1 mice aged 6 to 9 months were selected. First, the metabolic rate of brain glucose gradually decreased, as shown by PET/CT; the levels in 9-month-old APP/PS1 mice decreased more significantly. At 6 months of age, BBB disruption and neurological dysfunction could already be detected. Consistent with the previous results of reduced glucose metabolism detected by 18F-FDG positron emission tomography with AD-related encephalopathy [49, 50], these abnormal changes are most prominent in the fragile hippocampal structure and cortical areas. We also found that the decline in its metabolic rate was asymmetric, which may be related to the distribution and metabolic characteristics of Aβ in the brain. Moreover, the relationship between neurovascular integrity, brain structure and functional connectivity, cognitive function, and neurological symptoms, such as complex AD still needs to be directly explored in the most relevant in vivo environment. Western blot analysis of whole-brain protein samples showed that CD36 expression was reduced while as the expression of LRP1 was normal, which may indicate that CD36 was activated or inhibited in APP/PS1 mice, while LRP1 was not affected. CD36 is mainly expressed in macrophages and microglial cells, and CD36, as a receptor for microglial inflammation, has been confirmed in AD mouse models [27]. With regard to the decrease in CD36 protein expression in the whole brain, whether its relationship with pericytes affects the BBB is a new direction for future research.

In the present study, we found that the brain pericytes of APP/PS1 mice at the age of 6 months were reduced earlier than those of 18- to 22-month-old Tg2576 mice in the literature [29], suggesting that Aβ damage to pericytes may appear in AD. Early targeting and regulation of pericytes may represent a new therapeutic strategy for AD treatment in the early stage. This finding is consistent with a previous finding that excess Aβ in pericytes can induce cell death in human [51] and mouse [52] pericytes. Excessive Aβ deposition in pericytes often causes pericyte death during this accumulation, which will aggravate the pathological progression of AD. Here, we found that Aβ plaques formed and deposited in the blood vessels of 6-month-old APP/PS1 mice, indicating that the Aβ balance had been disrupted. Vascular transport is the fundamental way to clear Aβ from the brain [53]. In the early and late stages of AD, the clearance of β-amyloid appears to be impaired. Fluorescence results showed that PDGFRβ, Aβ, and CD36 are colocalized in the brain, indicating that pericytes could uptake Aβ through CD36. Moreover, the damage to the pericyte itself due to the removal of Aβ might also lead to the further development of capillary CAA [54, 55]. However, the response of CD36 on pericytes to Aβ and whether it affects the BBB require further research. We also explored whether CD36 is a potential therapeutic target to control Aβ level and clearance in AD. Therefore, in an in vitro study, we used low doses (100 nM and 1 µM) of Aβ1-40 to stimulate primary pericytes for 6 h and then conducted experiments. The study showed that CD36 expression was significantly higher than that in the control group. Previous studies have found that monocytes, microglia, platelets, and endothelial cells express high levels of CD36 [26, 56, 57], but few reports have described CD36 expression in pericytes. Our results indicated that CD36, as a phagocytic receptor, was present in pericytes. The decreased expression of CD36 in the brain (Fig. 1) of APP/PS1 mice may be related to the microglia and endothelial cells rich in CD36 content in the brain tissue. Here, we used pericyte and endothelial cell coculture to construct a BBB model. The TEER value was significantly higher than that in the endothelial cell culture model alone, indicating that pericytes played a pivotal role in the formation of the BBB. Our research showed that Aβ1-40 could aggravate BBB damage by binding to CD36 on the surface of pericytes, resulting in decreased TEER of the BBB and increased permeability after treatment with a low dose of Aβ1-40 for a short time. In addition, this treatment could lead to disruption of the BBB. Gene knockdown of CD36 expression alleviated the increase in BBB permeability and the disruption of compactness induced by Aβ1-40. This finding is consistent with the concept of restoration of microvascular function in APP mice lacking CD36 [29]. Our results are similar to the above findings, indicating that Aβ1-40 disrupts the integrity of the BBB through pericyte CD36 molecules. These findings affirm the previously unrecognized role of CD36 in the mechanism of vascular amyloid deposition, revealing that CD36 may be a potential therapeutic target for the BBB.

The degradation of Aβ in cells is usually carried out through three pathways: the ubiquitin–proteasome pathway, the autophagy-lysosomal pathway, and the endosome-lysosomal pathway—or Aβ is transported outside of the cell by exocytosis [58].Our study showed that Aβ1-40 inhibited pericyte proliferation in a time- and dose-dependent manner. Aβ1-40 (1 µM) caused a significant increase in mitochondrial ROS and decreased membrane potential, indicating that mitochondria were damaged. Damaged or redundant mitochondria are often degraded, and mitochondrial components can be recovered through mitochondrial autophagy [59, 60]. Expression of the mitochondrial inner membrane protein Tim23 and the matrix protein HSP60 decreased significantly after stimulation with 1 µM Aβ1-40, while the ratio of LC3II/LC3I increased significantly, indicating that Aβ induced an increase in mitochondrial autophagy [61, 62]. These results were confirmed by the autophagy agonist CCCP and inhibitor CQ combined with the mitochondrial inhibitor Mdivi-1, which showed that Aβ induced pericyte mitochondrial autophagy. Changes in the autophagic process play an essential role in the pathogenesis of many neurodegenerative diseases. Usually, Aβ is degraded in lysosomes due to high levels of autophagy. Nevertheless, in disease conditions, Aβ accumulates in many autophagosomes in dystrophic neurites and becomes the main intracellular reservoir of toxic peptides in the AD brain [63].Our results also showed that Aβ1-40 causes BBB disruption after stimulation of pericytes. To explore the mechanism of action, we tested the effect of Aβ1-40 on pericytes at the level of apoptosis. However, 1 µM Aβ1-40 did not cause pericyte apoptosis. Our findings are consistent with the results of a previous study [64], except that those authors used fibrinogen to stimulate pericytes, which initially activated autophagy but did not activate caspase 3 and/or kill pericytes in the early stage. Damaged mitochondria increase ROS production, and excessive ROS cause more damage to mitochondria [65, 66]. We found that Aβ1-40 increased ROS and decreased ATP production in pericytes, while SOD, which has antioxidant effects, did not exhibit increased expression.

ROS accumulate in an iron-dependent manner, leading to a new form of programmed necrosis called ferroptosis [42]. Ferroptosis is related to many diseases, including neurodegenerative diseases, acute renal failure, and cancer cardiomyopathy [67, 68]. We found that Aβ1-40 caused an increase in Fe2 + in pericytes, increased lipid ROS, and decreased GSH-Px. We further confirmed that Aβ1-40-induced ferroptosis occurred in pericytes by inhibiting GPx4 and xCT. GPx4 is a glutathione-dependent antioxidant enzyme that reduces membrane phospholipid hydroperoxide to inhibit ferroptosis [44, 68, 69]. The function of xCT is to import cysteine for glutathione biosynthesis and antioxidant defense [43]. Ferritin is an iron storage protein complex used to store excess iron. Many ROS-producing enzymes, such as nicotinamide adenine dinucleotide phosphate-oxidase (NOX), lipoxygenase, xanthine oxidase, and cytochrome P450 enzymes, contain iron in their active centers [46, 70]. In this experiment, Aβ1-40 stimulated pericytes, and no significant increase in NOX1 was found, indicating that NOX did not release Fe2 + . We also found that ferroptosis was blocked by Mdivi-1, suggesting that Aβ1-40 caused autophagy-dependent ferroptosis. We found that Aβ1-40 activated pericyte mitochondrial autophagy through the PINK1/Parkin pathway induced by PTEN. PINK1 is a mitochondrial-targeted serine/threonine kinase, and Parkin is a cytoplasmic ubiquitin E3 ligase. When mitochondria are damaged, the loss of mitochondrial membrane potential results in PINK1 accumulation on the outer mitochondrial membrane (OMM) [71]. PINK1 mediates the phosphorylation of ubiquitin serine 65 (Ser65) and equivalent serine residues in the ubiquitin-like domain of Parkin, leading to Parkin activation and recruitment [71–73]. Activated Parkin generates ubiquitin chains on OMM proteins [72] interact with the autophagosome resident protein LC3 [74, 75]. Then, the chains merge and fuse into a complete ring, separating each damaged mitochondrion into a mitochondrial autophagosome [30]. The OMM proteins NIP3-like protein X (NIP3-like protein X, NIX, also known as BNIP3L) and BCL2 interacting protein 3 (BNIP3) are related to mitochondrial morphology and mitochondrial autophagy [76, 77]. In this experiment, Aβ did not cause the activation of BNIP3/NIX. In addition, Parkin overexpression in AD mouse models led to enhanced autophagic clearance of defective mitochondria and prevented mitochondrial dysfunction [78, 79]. In short, among the many different mitochondrial autophagic pathways, PINK1/Parkin-dependent mitochondrial autophagy is the focus of current AD research. We found that after si-CD36 treatment, Aβ1-40-induced mitochondrial autophagy by PINK1/Parkin was blocked, indicating that Aβ1-40 caused pericyte mitochondrial autophagy through the CD36/PINK1/Parkin pathway.

## Conclusion

In summary, we discovered that PDGFRβ (a marker of pericytes), CD36, and Aβ colocalized in vitro and in vivo, and Aβ1-40 caused BBB disruption by upregulating CD36 expression in pericytes. The mechanism by which Aβ1-40 destroys the BBB involves the induction of pericyte mitophagy-dependent ferroptosis through the CD36/PINK1/Parkin pathway. In the future, more translational studies are needed to evaluate the role and mechanism of the regulation of mitochondrial autophagy and its induced ferroptosis for the treatment of aging, neurodegeneration, and other diseases.

## Supplementary Information

**Additional file 1:** **Figure S1.** Pericyte morphology and immunofluorescence identification. (A) Morphology of pericytes under a light microscope. Scale bar: 200 µm. (B) Immunofluorescence shows that these cells express PDGFRβ (red) but not the endothelial cell-specific protein CD31 (green). Blue (DAPI), scale bar: 50 µm.

## Data Availability

The datasets used and/or analyzed during the current study are available from the corresponding author on reasonable request.
