# Supplementary figures and images for: β-amyloid protein induces mitophagy-dependent ferroptosis through the CD36/PINK/PARKIN pathway leading to blood–brain barrier destruction in Alzheimer’s disease
Source: Cell Biosci. 2022 May 26;12:69. doi: 10.1186/s13578-022-00807-5 (PMC9134700; doi:10.1186/s13578-022-00807-5)

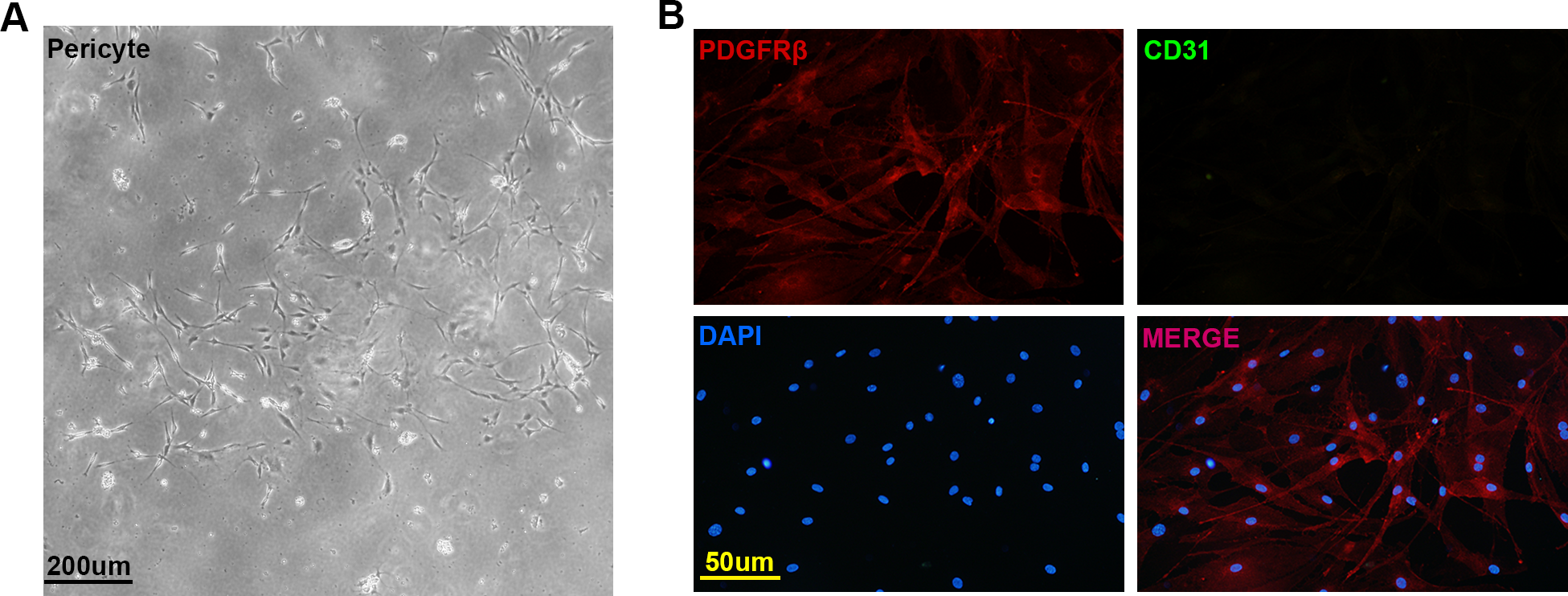

Supplement: Supplementary file 1 — Additional file 1: Figure S1. Pericyte morphology and immunofluorescence identification. (A) Morphology of pericytes under a light microscope. Scale bar: 200 µm. (B) Immunofluorescence shows that these cells express PDGFRβ (red) but not the endothelial cell-specific protein CD31 (green). Blue (DAPI), scale bar: 50 µm. [file 13578_2022_807_MOESM1_ESM.tif]
